# Occurrence of Occult Malignancies in Reduction Mammoplasties

**DOI:** 10.3389/fsurg.2018.00017

**Published:** 2018-02-28

**Authors:** Matthias Waldner, Holger J. Klein, Walter Künzi, Merlin Guggenheim, Jan A. Plock, Pietro Giovanoli

**Affiliations:** ^1^Division of Plastic Surgery and Hand Surgery, Universitäts Spital Zürich, Zurich, Switzerland; ^2^Euro-Policlinic Switzerland, Zurich, Switzerland; ^3^Swissparc Zürich, Zurich, Switzerland

**Keywords:** mammaplasty, breast cancer, histopathological analysis, retrospective studies, ductal carcinoma in situ (DCIS)

## Abstract

**Objectives:**

Patients undergoing reduction mammoplasty (RM) bear the risk of having occult breast cancer nests. The detection rate of malignant neoplasms in the resected specimens, varies greatly in the literature. The aim of our present study was to analyze risk factors and evaluate histopathological findings in our cohort of patients who underwent RM towards our center.

**Material and methods:**

In this retrospective single center study we analyzed 559 female patients [median age 35.99 (±13.34)] who underwent RM between 2000 and 2010. The presence of carcinoma and ductal- (DCIS) or lobular carcinoma in situ (LCIS) were considered as pathological findings. Body mass index (BMI), age, surgical technique and mass of resected tissue were included into the analysis.

**Results:**

There were 6 cases of occult neoplasia (1.08 %) including 2 cases of breast cancer, one multicentric DCIS and 3 cases of LCIS (0.54 %) in 559 patients. Patients with breast cancer showed a significant increased median age: 49y median (IQR ± 18) vs. 35y (IQR ± 21) (*p* = 0.004) and a trend towards increased BMI: 25.88 median (IQR ± 7.3) vs. 24.50 (IQR ± 4.09) (*p* = 0.219), compared to patients without pathological results. One patient with occult carcinoma had a negative preoperative mammography, a patient with LCIS a negative preoperative breast ultrasound.

**Conclusions:**

In our study the occurrence of occult neoplasia was associated with increased age and showed a trend towards increased BMI when compared to patients without pathological findings. The study demonstrates the necessity of thorough medical history, preoperative diagnostic screening and histopathological analysis of all resected specimens.

## Introduction

Breast reduction mammoplasty (RM) is a surgical intervention to reduce the volume and change the shape of the female breast. Indications include symptomatic macromastia, aesthetic reasons such as congenital or acquired asymmetry, or accompanying mastoptosis. The most common indication for breast reduction is symptomatic macromastia with patients suffering from neck pain, back pain, inframammary intertrigo and shoulder grooving. Different studies have shown the efficacy of mammoplasty for the treatment of symptomatic macromastia (1) with good long time results regarding pain relief and quality of life (2). For this reason, RM for symptomatic macromastia mostly takes part of third party reimbursement by health insurances. Recent studies showed therapeutic effects of RM on headache and migraines and suggested broader indication criteria (3). Other indications are congenital or acquired breast asymmetry, the latter mostly due to previous surgical interventions.

In the collective of patients undergoing breast reduction surgery, neoplastic changes of breast tissue are not primary expected. However, breast cancer is the most common malignant non-skin tumor in Swiss women (4) and the presence of occult breast cancer in reduction mammoplasty specimens has been described before (5–7). Therefore, the assessment of risk factors for the development of breast cancer and preoperative examinations (e.g., mammography) provide important data before surgery. In reduction mammoplasty, the presence of a contralateral breast cancer was found to be a major risk factor for the presence of occult carcinoma (8, 9). Different parameters to estimate the individual probability to develop malignant breast cancer are known (10) and should be part of every preoperative assessment of patients undergoing RM. Prior to surgery, information about age, age at menarche, age at first live birth and number of first-degree relatives with breast cancer should be assessed.

Between 2000 and 2010 different surgical techniques where described to make procedures simpler and to spare scarring (11–13) The aim of this study was to assess individual risk factors of patients with the finding of occult breast cancer and compare them to patients without malignant findings in reduction mammoplasties.

### Patients and Methods

This study was approved by the local ethics committee (KEK-ZH Nr.2011-0469). We reviewed the charts of 559 female patients, who underwent reduction mammoplasties between 2000 and 2010. Inclusion criteria were bilateral breast reduction mammaplasty and histological evaluation of resected specimens. Exclusion criteria were patients with a history of breast cancer or unilateral breast reductions. Preoperative screening reports were reviewed and patients with suspicious findings [BIRADS >3 (Breast Imaging Reporting and Data System)] excluded. Surgery was performed by different surgeons and using different surgical techniques. Surgical techniques included those described by Hall-Findlay (13), Lassus (11), Mc Kissock (14), Pitanguy (15) and Webster. Additional collected parameters were: age at surgery, BMI, preoperative mammography/ultrasound, reduction specimen weight and the presence of precancerous or malignant findings in the resected tissue.

All specimens were assessed by a board-certified pathologist. The tissue underwent macroscopic evaluation and dissection in 5 mm slices. Macroscopic suspicious areas underwent further microscopic analysis and processing. Results of histopathologic analysis were reviewed and diagnosis for carcinoma, LCIS or DCIS collected. Lobular hyperplasia was not considered.

### Statistical Analysis

Data was analyzed using Statistical Package for Social Sciences (SPSS, Version 20 for Macintosh; Chicago, Illinois). Discrete values were expressed as counts (percentages) and continuous variables as medians (IQR). Log10 transformation of age and BMI was employed to reach Gaussian distribution of data for parametric analysis. Univariate ANOVA was used to test for influence of age and diabetes on the occurrence of breast cancer. All tests were two tailed; *p* < 0.05 was considered significant.

## Results

**Figure 1:**
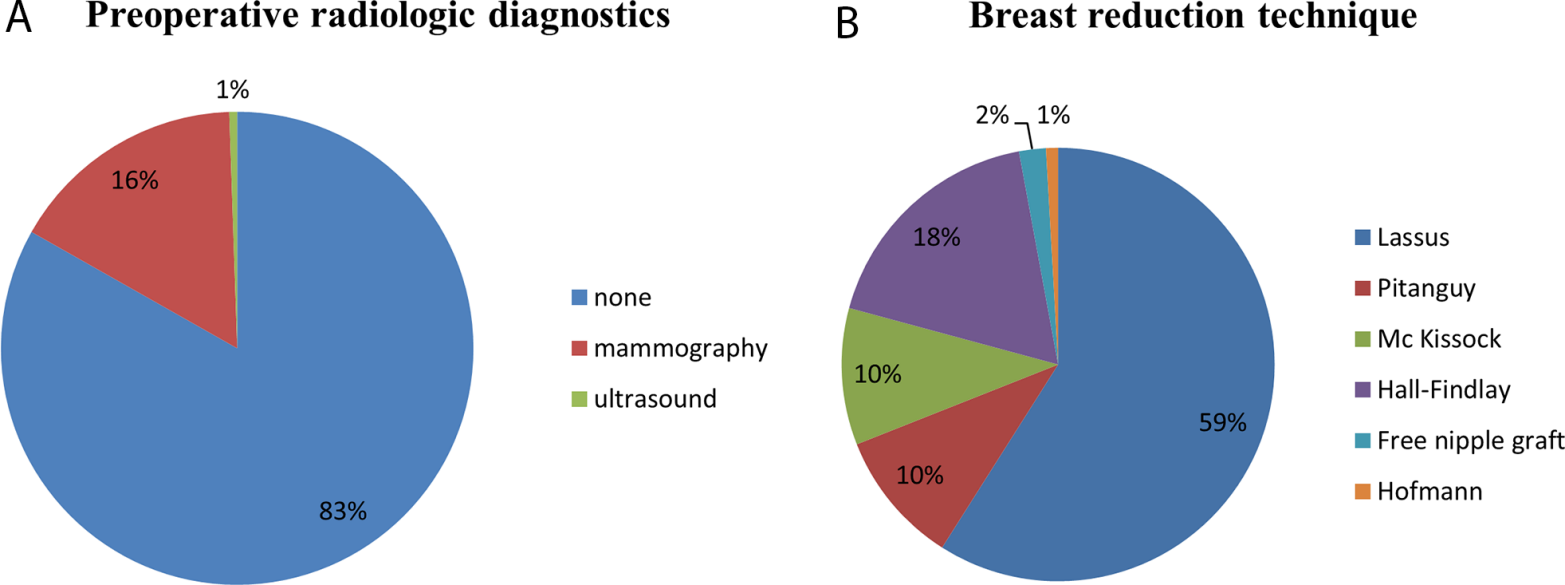
**(A)** Preoperative screening of patients undergoing reduction mammoplasties between 2000 and 2010. 16% of all patients underwent preoperative mammography, only 1% preoperative ultrasound of the breast. 83% had no preoperative radiologic diagnostic. **(B)** Demonstrates the surgical techniques applied, the majority of patients was operated according to the technique described by Lassus.

A total of 566 patients underwent RM between 2000 and 2010 of which 559 were included in this study. Three of the excluded patients had a history of breast cancer, 4 patients underwent unilateral procedures. The median BMI was 25.88 kg/m^2^, median age at surgery was 35 years. 16% of all operated patients underwent preoperative mammography, 0,5% preoperative ultrasound examination (Figure 1). None of the patients had suspicious findings in the preoperative mammography or ultrasound exceeding BIRADS 1. Surgical techniques performed were those described by Lassus (59%), followed by the Hall-Findlay (18%) and those described by Pitanguy (10%) and Mc Kissock (10%). Modified techniques described by Hofmann (0.9%) (16) or free nipple grafts (2%) (17) were performed less often. The mean resection weight was 481,5 (± 300.01 g/breast). Surgical technique and weight of the resected tissue was not associated with increased occurrence of malignant histological findings. 50 Patients (8.9%) were between the age 16 and 19 with the diagnosis of juvenile macromastia; none of these patients had malignant findings in their reduction specimen.

A total of 6 occult malignancies was found among the 559 patients included in the study. These findings included two cases of occult carcinoma, one multicentric DCIS and three findings of LCIS. Patient with findings of occult breast cancer showed a tendency towards increased BMI 25.88 median (IQR ± 7.3) vs. 24.50 (IQR ± 4.09) (*p* = 0.219), and a significant higher age: 49y median (IQR ± 18) vs. 35y (IQR ± 21) (*p* = 0.004) compared with patients without pathological findings. In all three patients with malignant findings, oncologic resection in form of conventional mastectomy (*n* = 2) or skin sparing mastectomy (*n* = 1) was performed, two patients underwent additional axillary lymphadenectomy. In all three patients an immediate autologous breast reconstruction was performed (Table 1). All three patients had a tumor-free follow up for the next 5 years (Table 1). Interestingly, patients number 2 and 3 (Table 1) had a preoperative mammography. In patient number 3 nodular mastopathy was describe without further suspicious lesions, in patient number 2 the mammography showed no suspicious findings. The three patients with a diagnosis of LCIS were closely observed using radiologic and clinical evaluation. None of these patients developed breast cancer in the following 4 years. The follow up consisted in chart reviews of 4 years after surgery.

**Table 1:** Malignant findings among resection mammoplasty patients and oncologic surgery

	**Age**	**BMI**	**Diagnosis**	**Oncologic surgery**	**Breast reconstruction**
1	50–55	33.5	Adeno carcinoma (r), Stage 2a	Mastectomy, LAD (r)	Pedicled lat. dorsi flap (r)
2	45–50	33.6	Multic. DCIS (b), Stage 1a	s.s.Mastectomy (b)	Free m. gracilis flap (b)
3	60–65	29.8	Ductal carcinoma (r)	Mastectomy, LAD (r)	Pedicled lat. dorsi flap (r)

(r) right, (b) bilateral. Patients one and three underwent axillary lymphadenectomy (LAD) and conventional mastectomy, while in patient number 2 a skin sparing mastectomy was performed. All patients had a tumor free follow up.

## Discussion

In our study, the rate of occult malignancies among 559 patients undergoing RM was 1.08% over a period of 10 years. A number of studies over the past years showed variable rates of occult breast cancer in RM, varying from 0.03 to 5.45% (6, 7, 18–20). The variance of these results is probably the consequence of different methodologies and study designs. Some studies treat the LCIS as a separate group of histopathologic findings in RM, representing a risk factor for development of invasive cancer in either breast (8). In spite of these facts, trends in the distribution of pathologic findings among patients can be detected. In this study patients with malignant findings were significantly older than patients without malignancies. This correlates with findings Ambaye et al. (5) were almost all malignant occult malignancies were found in patients older than 40 years of age. In a subgroup analysis by Hassan and Pacifico (18) all significant findings were detected in patients over 29 years old with an increasing risk with age. This study included patients undergoing symmetrizing mammoplasty after contralateral breast cancer, a group we excluded in our study.

In our study patients with breast cancer had a tendency towards a higher BMI compared to patients without malignant findings. There is evidence for a relationship between increased risk for breast cancer, especially for postmenopausal patients with increased BMI (21, 22). A prospective analysis of 103.344 postmenopausal women by Lahmann et al. (23) demonstrated association between weight, BMI and hip circumference in postmenopausal patients without hormone replacement therapy. This trend was not observed in premenopausal woman. These findings line out that postmenopausal woman with increased weight should be thoroughly assessed preoperatively.

In breast reduction surgery, the histopathologic assessment of resected breast tissue specimens is still subject of debate (18). Resected specimens are often fragmented and do not always have orientation markings. The appearance of the resected tissue is dependent on the surgical technique, the surgical team and additional intraoperative resections, to obtain a good shape and symmetric results of the breast. The surgical technique should be clearly defined for the pathologist to improve orientation on the specimen. In contrast to the defined histopathological guidelines (24) for the analysis of skin sparing mastectomy specimens, or conventional mastectomy specimens, there are no clear rules how to mark and assess reduction mammoplasty specimens. The focus of the histopathologic examination is commonly influenced by macroscopic suspicious areas in the resected tissue. Histological analysis thus comprises commonly exemplary areas of the specimen and not its entirety. Ambaye et al. (5) showed that increased sampling and additional histopathologic sections lead to higher detection rates of pathologic findings in RM specimens, but are associated with higher costs. These costs could be reduced by limiting extra sections on patients with a high-risk profile for the presence of occult malignancies. Precise marking and orientation of the resected RM specimens by the surgeon allows the pathologist to localize pathologic findings more adequately. An additional benefit of additional anatomical marking is the potential prevention of lumpectomy, allowing for breast conserving therapy in patients with low stage tumors (25).

The role of tobacco smoking in the development of breast cancer remains unclear. Some authors described smoking cessation to be associated with an increase in risk of breast cancer relative to that in current smokers, and suspect a “antiestrogenic” or other effects of tobacco smoke, which is reversible after exposure ends (26, 27). In controversy, a subgroup analysis of patients with elevated risk, showed that smoking has an impact on breast cancer risk (28) History of breast cancer among first- degree relatives can give important information to detect BRCA1 or BRCA2 mutations as well as other inherited genetically determined risk factor such as p53 mutations. Patients with BRCA1 and BRCA2 mutations bear a risk for the development of breast cancer up to 80% (29). Ethnicity, weight and tall stature, where found to be modifying the individual risk for the development of breast cancer (30), but are not subject of standard preoperative assessment.

Preoperative radiologic evaluation is an important tool to reduce the number of occult carcinomas prior to surgery. In young patients the sensitivity of conventional mammography, especially in patients with very dense breasts, is reduced (31, 32). A higher accuracy for breast cancer detection by 3D digital mammography compared to the conventional technique, especially in woman under 50 years of age with fibro-glandular breast could be shown (33). Adjunctive breast- ultrasound has shown increased screening sensitivity, even though more - false positive findings result (34). Patients with a known elevated familial risk to develop breast cancer, benefit from a significantly higher sensitivity of MRI screening compared to mammography even if combined with ultrasound diagnostics (35). In our study, two patients with occult malignant findings demonstrated no suspicious lesions in the preoperative mammography. Campbell et al. (36) analyzed the role of preoperative mammography in woman seeking reduction mammoplasty and noted a high incidence of false positive results, without malignant findings in the specimen. Although, the incidental discovery of atypical neoplasia or lobular carcinoma in their study, did not significantly correlate with an abnormal preoperative mammographic result.

In conclusion, a thorough preoperative assessment to identify patients with elevated risks is important. This should include medical and family history, physical examination and radiologic diagnostics dependent on breast density and predisposition in order to reduce the number of occult beast malignancies preoperatively. A precise orientation of resected breast tissue and standardized histopathologic evaluation are necessary for diagnosis and staging in case of occult carcinoma. Limitations of this study are the retrospective study design, and the incomplete assessment of concomitant risk factors such as smoking, and information about family history.

## Ethics

This retrospective study was approved by the Kantonale Ethikkommission Zürich Approval number: KEK-ZH Nr.2011-0469

## Author Contributions

MW wrote the manuscript, collected data, HK performed statistical analysis, WK collected data, MG wrote part of the ethics application and part of the manuscript, JP reviewed and edited the manuscript, PG helped with ethics approval, reviewed the manuscript

## Conflict of Interest Statement

The authors declare that the research was conducted in the absence of any commercial or financial relationships that could be construed as a potential conflict of interest.
